# Age-dependent sensitization of cutaneous nociceptors during developmental inflammation

**DOI:** 10.1186/1744-8069-10-34

**Published:** 2014-06-07

**Authors:** Michael P Jankowski, Jessica L Ross, Jonathon D Weber, Frank B Lee, Aaron T Shank, Renita C Hudgins

**Affiliations:** 1 Department of Anesthesia, Division of Pain Management, Cincinnati Children’s Hospital Medical Center, 3333 Burnet Ave MLC 6016, Cincinnati, OH 45229, USA; 2 Department of Pediatrics, University of Cincinnati, Cincinnati, OH 45229, USA

**Keywords:** Neonate, Inflammation, Primary afferents, Electrophysiology, Molecular biology, Plasticity

## Abstract

**Background:**

It is well-documented that neonates can experience pain after injury. However, the contribution of individual populations of sensory neurons to neonatal pain is not clearly understood. Here we characterized the functional response properties and neurochemical phenotypes of single primary afferents after injection of carrageenan into the hairy hindpaw skin using a neonatal *ex vivo* recording preparation.

**Results:**

During normal development, we found that individual afferent response properties are generally unaltered. However, at the time period in which some sensory neurons switch their neurotrophic factor responsiveness, we observe a functional switch in slowly conducting, broad spiking fibers (“C”-fiber nociceptors) from mechanically sensitive and thermally insensitive (CM) to polymodal (CPM). Cutaneous inflammation induced prior to this switch (postnatal day 7) specifically altered mechanical and heat responsiveness, and heat thresholds in fast conducting, broad spiking (“A”-fiber) afferents. Furthermore, hairy skin inflammation at P7 transiently delayed the functional shift from CM to CPM. Conversely, induction of cutaneous inflammation after the functional switch (at P14) caused an increase in mechanical and thermal responsiveness exclusively in the CM and CPM neurons. Immunocytochemical analysis showed that inflammation at either time point induced TRPV1 expression in normally non-TRPV1 expressing CPMs. Realtime PCR and western blotting analyses revealed that specific receptors/channels involved in sensory transduction were differentially altered in the DRGs depending on whether inflammation was induced prior to or after the functional changes in afferent prevalence.

**Conclusion:**

These data suggest that the mechanisms of neonatal pain development may be generated by different afferent subtypes and receptors/channels in an age-related manner.

## Background

The contribution of sensory neurons to pain development has been extensively documented in adult animals (refs rev. in [1]). However, the role that sensory neurons play in pain development during early life is not as well understood. Many studies have analyzed sensory neurons in developing animals, but have typically analyzed dissociated neurons in response to various stimuli [2-8], or studied the changes in neurochemical or anatomical aspects of dorsal root ganglia (DRGs; [9-13]) in relation to pain behaviors [14,15] after injuries.

Some studies have evaluated sensory function with *in vitro* or *in vivo* systems in neonates [16-21]. These data have suggested that in uninjured mice, the individual subpopulations of sensory neurons mimic the functional response properties of their adult equivalents. Thus it could be suggested that sensitization of sensory neurons in neonates in response to peripheral damage would be similar as adults. However, it is well known that target derived neurotrophic factors are vital for the proper development of primary afferents and many neonatal sensory neurons switch their neurotrophic factor responsiveness from nerve growth factor (NGF) to glial cell line-derived neurotrophic factor (GDNF) during postnatal development [22-24]. Specifically, NGF has been shown to be crucial for the development of myelinated nociceptors [25-27] and may play a role in C-fiber heat responsiveness [28]. GDNF signaling has also been linked to the development of heat responsiveness in mouse sensory neurons [29], and may in fact be regulated by early NGF signaling [22]. In addition, it has been demonstrated that there is an early postnatal loss of heat sensitivity in myelinated sensory neurons during the first two weeks of life [30].

Therefore, injury at different stages of postnatal development may actually lead to distinct changes in sensory neurons compared to what is observed in adult animals. In support of this notion, one study that analyzed the functional response properties of developing sensory neurons *in vivo* during skin incision [31] showed that there were differences in mechanical sensitivity during neonatal injury that were not observed in older mice [32]. Nevertheless, there has not been a comprehensive analysis of the plasticity that occurs in sensory afferents after injury induced at different stages of postnatal development that span the period in which sensory afferents are thought to experience normal phenotypic switching [22,24]. We thus first sought to functionally define sensory neurons during this critical period of postnatal development in order to determine whether there were any functional correlates to known changes in afferent phenotype. Then we tested the hypothesis that peripheral injury during early life and prior to these phenotypic switches uniquely alters sensory neuron responsiveness compared to injury following the second week of life and after the observed switching. To test this, we used a neonatal *ex vivo* recording preparation to analyze the comprehensive phenotypes of individual afferents in mice with peripheral inflammation induced at either P7 or P14 by injection of carrageenan into the hairy hindpaw skin. Changes in afferent function were then compared to changes in mRNA and protein expression in the DRGs to determine potential mechanisms for the observed alterations in function. We have found that hairy skin inflammation induced at P7 distinctly alters sensory neuron responsiveness with corresponding upregulation of specific receptors/channels in the DRGs involved in sensory transduction compared to P14 inflammation. P7 inflammation specifically was also found to delay normal changes in sensory neuron prevalence at the same time they are thought to undergo phenotypic switches [22,24].

## Results

### Response properties of cutaneous afferents during postnatal development

**Figure 1:**
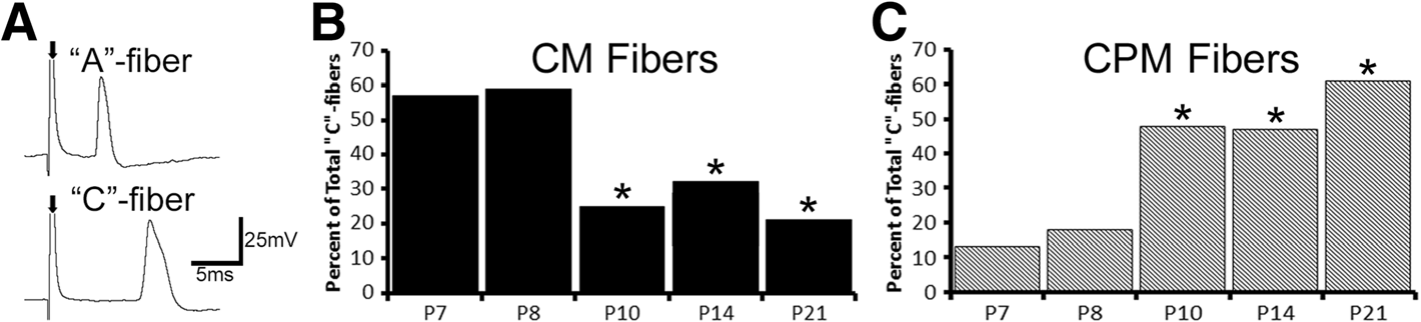
**Percent of total slowly conducting, broad spiking afferents. A**: Examples of action potentials generated from a fast conducting, broad spiking afferent (“A”-fiber) and a slowly conducting, broad spiking afferent (“C”-fiber) during development. From the onset of the electrical stimulus (arrows), the initiation of the “A”-fiber action potential was approximately twice as fast as the “C”-fiber potential. **B**: The percentage of mechanically sensitive, but thermally insensitive “C”-fibers (CM) out of all C-fibers recorded were significantly reduced beginning at postnatal day 10 (P10). **C**: Conversely, the percentage of mechanically sensitive and heat sensitive (and sometimes cold/cooling responsive) “C”-fiber afferents (CPM) was significantly increased beginning at P10. *p value < 0.05, *χ*^2^.

To confirm earlier reports (e.g. [19,21,30]) and first determine any age-related changes in peripheral response properties of individual cutaneous afferents in neonatal mice, we performed single unit recording with our neonatal *ex vivo* hairy skin/saphenous nerve/DRG/spinal cord preparation in uninjured mice from postnatal day seven (P7) through P21 (*seeMethodsfor information on animal numbers and average afferents recorded per group/condition*). Two clear categories of sensory afferents were detected based on conduction velocity (CV) within each experiment. Fast conducting “A”-fibers had at least double the CVs as slowly conducting “C”-fibers in each preparation (Figure 1A; [30]). Overall, “C”-fibers had a mean CV = 0.53 m/s. (<P10 Ave. = 0.54 m/s; ≥P10 Ave. = 0.53 m/s) while “A”-fibers had a mean CV = 3.26 m/s (<P10 Ave. = 2.01 m/s; ≥P10 Ave. = 3.72 m/s). These two CV classifications could then be further subdivided based on spike width (narrow vs. broad) and then separated based on response characteristics. Both fast (“A”-fibers) and slowly (“C”-fibers) conducting, narrow spiking afferents were found to have no differences in peripheral response properties (mechanical or thermal) at any of the ages tested (not shown), including after inflammation, similar to previous reports [33]. These included cells with “A”-fiber CVs that displayed rapidly adapting and slowly adapting type I responses, and “C”-fibers that were either mechanically sensitive and cold/cooling sensitive or mechanically insensitive and cold/cooling sensitive. Therefore, the remainder of this report will focus on the broad spiking afferent subclasses (putative nociceptors).

#### ***Naïve A-fibers***

When specifically analyzing the afferents with fast CVs and broad spikes, we found that the responses to mechanical stimuli were not different between the mechanically sensitive, thermally insensitive (AM), and mechanically and heat (and sometimes cold) sensitive (polymodal) fibers (APM); therefore, data analyzing response properties from these cell types was combined for ease of presentation hereafter. The AMs/APMs displayed no overt changes in mechanical thresholds (P7: 16.3 mN ± 9.2 mN; P8: 55 mN ± 45 mN; P10: 75 mN ± 25 mN; P14: 21.7 mN ± 14.2 mN; P21: 33.5 mN ± 22.1 mN; p value < 0.3) or mean peak instantaneous frequencies (P7: 75.8 Hz ± 20.4 Hz; P8: 40 Hz ± 18.5 Hz; P10: 65.56 Hz ± 32.4 Hz; P14: 110.7 Hz ± 103.9 Hz; P21: 41.4 Hz ± 16.9 Hz; p value < 0.63) to deformation of the skin (P7: n = 6; P8: n = 5; P10: n = 3; P14: n = 3; P21: n = 6). There were also no changes in those cells with “A”-fiber CVs that responded to heat (P7: n = 3; P8: n = 1; P10: n = 1; P14: n = 2; P21: n = 1) in terms of the thresholds (P7: 48.4°C ± 2.7°C; P8: 44.4°C ± 0°C; P10: 45.7°C ± 0°C; P14: 42.6°C ± 5.3°C; P21: 42.7°C ± 0°C) or firing (P7: 4.6 Hz ± 2.8 Hz; P8: 1.9 Hz ± 0 Hz; P10: 1.7 Hz ± 0 Hz; P14: 1.5 Hz ± 1.3 Hz; P21: 9.2 Hz ± 0 Hz; p value < 0.75) to heat stimuli at any developmental age tested.

Since we obtained low numbers of heat sensitive afferents at some of these developmental time points in naïve mice precluding us from an accurate statistical analysis across ages, we also combined data from *ex vivo* experiments in mice < P14 (P7-P10) and those ≥ P14 (P14 and P21) to at least determine if there were any changes in heat sensitivity over a larger time period during development. Combined data from these groups again shows no differences in heat thresholds (<P14: 47.1°C ± 1.7°C; n = 5 vs. ≥P14: 42.6°C ± 2.3°C; n = 3; p value < 0.2) or firing to heat stimuli (<P14: 3.5 Hz ± 1.7 Hz vs. ≥P14: 4.0 Hz ± 2.7 Hz; p value < 0.88). These results are consistent with previous reports showing a lack of changes in A-fiber response properties during this period of postnatal development [19,21,30]. Finally, only one broad spiking “A”-fiber (detected at P14) had a cold/cooling response out of all naïve “A”-fibers recorded.

#### ***Naïve C-fibers***

Similar results were found in the slowest conducting (“C”-fibers), broad spiking afferents that responded to mechanical and heat stimuli and sometimes cold (“C”-polymodal; CPM; P7: n = 3; P8: n = 6; P10: n = 12; P14: n = 14; P21: n = 22). No changes in mechanical thresholds (P7: 21.7 mN ± 14.2 mN; P8: 18 mN ± 11.1 mN; P10: 8.3 mN ± 3.9 mN; P14: 22.3 mN ± 8.7 mN; P21: 8.5 mN ± 1.8 mN; p value < 0.33) or firing (P7: 52.2 Hz ± 27.7 Hz; P8: 74.2 Hz ± 16 Hz; P10: 38.9 Hz ± 7.2 Hz; P14: 40.7 Hz ± 12.7 Hz; P21: 56.5 Hz ± 6.3 Hz; p value < 0.24) to mechanical stimuli were observed in these fibers over time. In addition, the responsiveness to heat stimuli in these afferents were not different over time (Heat Thresholds: P7: 47.2°C ± 2.5°C; P8: 43.5°C ± 2.7°C; P10: 44.5°C ± 1.4°C; P14: 42.1°C ± 1.6°C; P21: 43.2°C ± 0.8°C; p value < 0.5; Heat Instantaneous Frequencies: P7: 5.0 Hz ± 2.7 Hz; P8: 3.7 Hz ± 1.4 Hz; P10: 17.0 Hz ± 7.9 Hz; P14: 11.2 Hz ± 3.8 Hz; P21: 13.2 Hz ± 3.1 Hz; p value < 0.52). Of the CPM neurons that displayed a cold/cooling response (P7: n = 1; P8: n = 0; P10: n = 3; P14: n = 3; P21: n = 13), no differences were observed over time for these response properties (Cold/Cooling Thresholds: P7: 8.9°C ± 0°C; P8: undetermined; P10: 21.3°C ± 2°C; P14: 10.8°C ± 1.6°C; P21: 11.8°C ± 1.2°C; Cold/Cooling Instantaneous Frequencies: P7: 1.5 Hz ± 0Hz; P8: undetermined; P10: 3.1 Hz ± 1.5 Hz; P14: 3.1 Hz ± 1.9 Hz; P21: 4.5 Hz ± 2.4 Hz). Similar results were obtained if we combined these data into groups < P14 and ≥ P14 (Cold Thresholds: 13.9°C ± 2.1°C vs. 11.6°C ± 1.0°C, n = 4; Cold Firing: 2.1 Hz ± 0.3 Hz vs. 4.0 Hz ± 0.7 Hz, n = 16; p value < 0.4).

The mechanically insensitive, heat sensitive “C”-fiber afferents (CH; P7: n = 2; P8: n = 2; P10: n = 2; P14: n = 4; P21: n = 3) also showed no differences in heat threshold (P7: 44.8°C ± 0.1°C; P8: 43.0°C ± 0°C; P10: 42.9°C ± 0.4°C; P14: 44.7°C ± 2.2°C; P21: 42.8°C ± 2.9°C; p value < 0.92) or firing (P7: 19.1 Hz ± 17.7 Hz; P8: 20.0 Hz ± 0 Hz; P10: 42.8 Hz ± 41.7 Hz; P14: 30.5 Hz ± 8.3 Hz; P21: 9.8 Hz ± 3.8 Hz; p value < 0.76) to heat stimuli at all ages tested. Combining these data into < P14 (n = 6) and ≥ P14 (n = 7) also revealed no changes over time (Heat Thresholds: 43.5°C ± 0.4°C vs. 43.9°C ± 1.6°C, p value < 0.76; Heat Firing: 27.3 Hz ± 12.7 Hz vs. 23.6 Hz ± 6.9 Hz, p value < 0.8). However, one cell type, the mechanically sensitive, thermally insensitive fibers (CM; P7: n = 11; P8: n = 15; P10: n = 5; P14: n = 8; P21: n = 7), showed reduced firing to mechanical stimulation of the skin beginning at P10 (26.9 Hz ± 7.9 Hz; p value < 0.05) relative to both P7 (74.6 Hz ± 8.0 Hz) and P8 (50.6 Hz ± 9.8 Hz) mice. This trend continued at P14 (25.0 Hz ± 7.6 Hz; p value < 0.05) and P21 (18.3 Hz ± 7.5 Hz; p value < 0.05). The mechanical thresholds in these afferents however, were not found to be altered at any age tested (P7: 21.0 mN ± 9.7 mN; P8: 50.6 mN ± 11.7 mN; P10: 48.3 mN ± 25.9 mN; P14: 53.3 mN ± 3.33 mN; P21: 60.6 mN ± 18.2 mN; p value < 0.25).

### Percentages of cutaneous afferents during postnatal development

We then wanted to determine if there were changes in the total numbers of any afferent types recorded at the different postnatal ages analyzed. Although there were no differences in the percentage of CH neurons observed out of all C-fibers detected during recordings (P7: 13%, n = 4/30; P8: 5%, n = 2/44; P10: 7%, n = 3/44; P14: 11%, n = 4/38; P21: 9%, n = 4/44), we did observe a shift in the percentage of both the CM and CPM fibers recorded between P8 and P10. At P7 (57%, n = 17/30) and P8 (59%, n = 26/44), the majority of slowly conducting broad spiking afferents were found to be of the CM subtype; however, the percentage of these afferents detected beginning at P10 (25%, n = 11/44; p value < 0.05) was significantly reduced (Figure 1B). This was maintained at both P14 (32%, n = 12/38; p value < 0.05) and P21 (21%, n = 9/44; p value < 0.05). Corresponding with the reduction in CM fiber prevalence at these time points was a significant increase in the percentage of CPM fibers (Figure 1C). At P7 (13%, n = 4/30) and P8 (18%, n = 8/44), few fibers were found to be of the CPM classification, but at P10 (48%, n = 21/44; p value < 0.05), there was a significant increase in CPM neuron prevalence, which was maintained at the later developmental time points (P14: 47%, n = 18/38; P21: 61%, n = 27/44; p values < 0.05).

The percentage of AMs and APMs out of all A-fibers recorded; however, showed no differences at any postnatal age tested (AM: P7: 28%, n = 5/18; P8: 40%, n = 4/10; P10: 43%, n = 3/7; P14: 23%, n = 3/13; P21: 42%, n = 5/12; p value < 0.78; APM: P7: 17%, n = 3/18; P8: 10%, n = 1/10; P10: 14%, n = 1/7; P14: 15%, n = 2/13; P21: 8%, n = 1/12; p value < 0.98). Taken together these data suggest that the response properties of *individual* saphenous afferent subtypes are generally not different during postnatal development, consistent with previous reports utilizing similar cutaneous preparations [19,30]. However, unlike “A”-fiber subpopulations which appear to display differences in frequency within the first week of life [30], the prevalence of “C”-fiber subtypes changes significantly during the second week of life (between P8 and P10).

### Gene expression patterns in DRGs during postnatal development

In order to determine potential mechanisms for the changes in afferent response properties and prevalence during early development, we performed realtime PCR on the L2/L3 DRGs from P7 through P21 for a variety of receptors/channels known to be involved in sensory transduction (Thermo-TRP channels, acid sensing ion channels (ASIC), purinergic receptors (P2Y1 and P2X3) and Piezo channels) or signaling from the periphery (neurotrophic factor and cytokine receptors). Results from these time points were compared to L2/L3 DRGs acquired at P0 to determine how expression changed during the course of development (n = 3-5 per condition/age). In general, the relative expression of most genes was unaltered during development (Table 1) consistent with results obtained from *ex vivo* recording. However, some genes were found to be altered beginning at postnatal day 10, which we have shown to be the time point at which “C”-fiber afferents show significant changes in fiber frequency between the CM and CPM subtypes (Figure 1), and in the firing of CM neurons to mechanical stimuli (above). As a first step in our analysis however, we first wanted to verify previous reports (e.g. [22,24]) that there was a decrease in the nerve growth factor (NGF) receptor trkA in the DRGs during this period. This would also help us confirm the veracity of our data obtained from the various age groups. We indeed confirmed that relative to P0, there was a significant reduction in trkA mRNA in the DRGs at P7 (-55 ± 8%; p value < 0.05). This remained decreased in the DRGs at all developmental time points thereafter (P8 to P21). Since a switch in neurotrophic factor responsiveness in sensory neurons is thought to occur between NGF and glial cell line-derived neurotrophic factor (GDNF) to modulate many processes during normal development [22,24,25,27,29], we then analyzed the expression of the GDNF co-receptor GFRα1 in the DRGs at these developmental time points. We found a significant increase in the levels of this neurotrophic factor co-receptor at P10 (75 ± 29%; p value < 0.05) and the levels of this co-receptor remained elevated thereafter (Table 1).

**Table 1:** Percent changes in mRNA expression in the L2/L3 DRGs of naïve mice

**Gene**	**Postnatal Day 7**	**Postnatal Day 8**	**Postnatal Day 10**	**Postnatal Day 14**	**Postnatal Day 15**	**Postnatal Day 17**	**Postnatal Day 21**
trkA	-55 ± 8*	-57 ± 14*	-63 ± 11*	-74 ± 12*	-72 ± 13*	-78 ± 18*	-84 ± 21*
GFRα1	1 + 20	56 ± 31	75 ± 29*	101 ± 33*	102 ± 41*	49 ± 7*	67 ± 23*
GFRα3	-43 ± 8	-44 ± 15	-58 ± 8*	-72 ± 13*	-71 ± 12*	-71 ± 12*	-76 ± 21*
IGFr1	13 ± 35	14+ ± 5	-7 ± 29	-30 ± 29	-52 ± 13	34 ± 29	-40 ± 24
IL1r1	115 ± 20*	70 ± 21*	53 ± 15*	68 ± 44	62 ± 18*	109 ± 20*	-44 ± 20
ASIC1	-7 ± 24	-14 ± 28	-20 ± 21	-35 ± 18	57 ± 34	-27 ± 19	-45 ± 23
ASIC3	-3 ± 31	-38 ± 29	4 ± 27	-8 ± 27	-21 ± 15	-7 ± 21	-17 ± 24
TRPA1	885 ± 30*	1312 ± 29*	2196 ± 17*	2517 ± 19*	1639 ± 53*	3014 ± 17*	2442 ± 23*
TRPC3	53 ± 9*	57 ± 12*	27 ± 10	-22 ± 10	-41 ± 13	11 ± 19	7 ± 16
TRPM3	-4 ± 20	-44 ± 18	-33 ± 16	-53 ± 17*	-59 ± 23*	-57 ± 17*	-58 ± 18*
TRPM6	206 ± 26*	95 ± 23*	198 ± 18*	104 ± 25*	102 ± 26*	330 ± 27*	240 ± 20*
TRPV1	111 ± 16*	60 ± 20^#^	60 ± 19^#^	36 ± 23	34 ± 15	25 ± 25	35 ± 28
P2Y1	60 ± 22^#^	28 ± 24	80 ± 30	32 ± 22	35 ± 24	42 ± 20	22 ± 20
P2X3	-7 ± 11	-16 ± 15	-44 ± 7*	-50 ± 10*	-64 ± 13*	-59 ± 6*	-57 ± 18*
Piezo2	25 ± 19	34 ± 19	-17 ± 22	-30 ± 22	-53 ± 18^#^	-33 ± 15	-31 ± 22

Data presented are percent changes in expression from DRGs obtained at postnatal day 7 through postnatal day 21 relative to naïve P0 DRGs. *p value < 0.05; #p value < 0.1. Mean ± SEM; One-way ANOVA with Tukey’s post hoc test.

At this same time point (P10), we also found a significant decrease in P2X3 (-44 ± 7%; p value < 0.05) and the artemin neurotrophic factor co-receptor GFRα3 (-58 ± 8%; p value < 0.05). Prior to this time point, there was also significantly increased mRNA levels of the excitability regulator TRPC3 at P7 (53 ± 9%; p value < 0.05) and P8 (57 ± 12%; p value < 0.05) compared to P0 DRGs; however, levels were not found to be different than P0 expression from P10 onward. Finally, we found a significant increase in TRPV1 expression, but only at P7 (111 ± 16%; p value < 0.05). Other interesting changes in expression detected during the first three weeks of life included a significant increase in the Mg^2+^ channel TRPM6 [34,35] at P7 (206 ± 26%; p value < 0.05) and all other time-points tested. In addition, we found a highly significant increase in the extreme cold responsive and irritant receptor TRPA1 [36,37] at P7 (885 ± 30%; p value < 0.05), and this also remained highly elevated thereafter.

### Age-related changes in response properties of cutaneous afferents during neonatal inflammation

Since we observed no changes in “A”-fiber prevalence or response properties during the second and third weeks of life, but did observe changes in CM neuron firing and the prevalence of CM and CPM neurons between P8 and P10, we wanted to determine if there were age-specific changes in the response properties of individual afferent subtypes after cutaneous inflammation initiated at time points that spanned this critical period of postnatal sensory neuron development. We therefore performed *ex vivo* recording analysis in mice one, three and seven days after inflammation of the hairy hindpaw skin induced at either P7 or P14 by injection of 3% carrageenan. We first determined paw edema in these mice to ensure that a similar degree of inflammation was achieved between the different age groups. At P7, the average paw volume ratio between the ipsilateral and contralateral hindpaws after carrageenan injection was 2.0 ± 0.1 at 1d, 1.6 ± 0.1 at 3d and 1.3 ± 0.1 at 7d post inflammation.

#### ***P7 Inflamed A-fibers***

**Figure 2:**
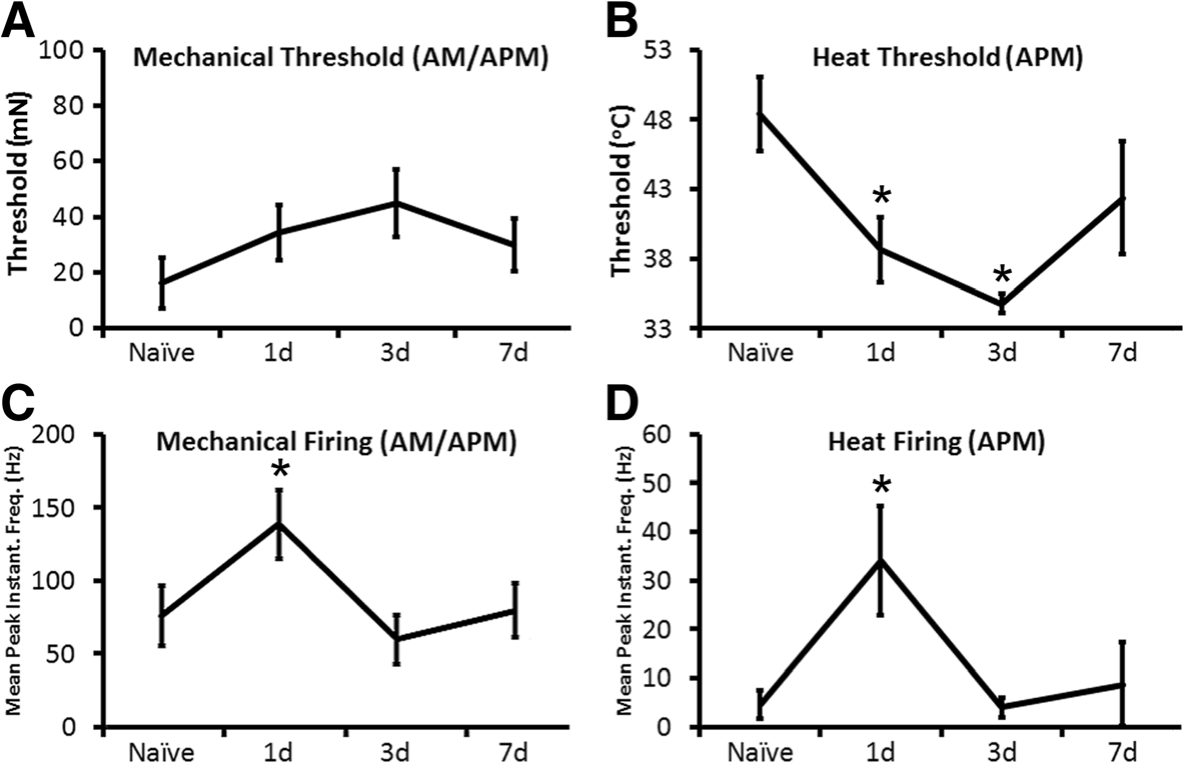
**Response properties of fast conducting, broad spiking afferents after cutaneous inflammation initiated at P7. A**: No changes in mechanical thresholds were found in the fast conducting, broad spiking afferents that were mechanically sensitive (AM) or mechanically and heat sensitive (APM) at any time point after inflammation of the hairy skin initiated at postnatal day 7 (P7). **B**: However, the heat thresholds of the APM neurons were found to be significantly reduced one and three days after P7 inflammation. Heat thresholds returned to naïve levels by 7d post hairy skin inflammation. **C**: The mean peak instantaneous frequencies to mechanical deformation of the skin were also found to be significantly increased one day after P7 inflammation in the AM/APM neurons. **D**: In addition, the mean peak instantaneous frequencies to heat stimuli were enhanced in APMs one day after cutaneous inflammation initiated at P7. *p value <0.05, mean ± SEM; One-way ANOVA with Tukey’s post hoc test for thresholds and Kruskal-Wallis and Dunn’s post hoc test for instantaneous frequencies. See text for animal and afferent numbers.

No differences were found between AM and APM fibers in terms of their responsiveness to mechanical stimuli after inflammation (Naive: n = 6; 1d: n = 16; 3d: n = 10; 7d: n = 6) and therefore are combined for ease of presentation. We found one day after cutaneous inflammation induced at P7 that the AM/APM fibers had significantly higher firing to mechanical stimuli (75.8 Hz ± 20.4 Hz vs. 138.6 Hz ± 23.7 Hz; p value < 0.05) but this returned to naïve levels by day three (Figure 2C). No changes in mechanical thresholds (Naïve: 16.3 mN ± 9.2 mN; 1d: 34.5 mN ± 9.9 mN; 3d: 44.9 mN ± 12.2 mN; 29.8 mN ± 9.5 mN; p value < 0.38); however, were observed at any time point after P7 inflammation in these cells (Figure 2A). The APM fibers also had significantly higher mean peak instantaneous frequencies to heat stimulation of the skin (Naive: n = 3; 1d: n = 7; 3d: n = 3; 7d: n = 2) at one day (4.6 Hz ± 2.8 Hz vs. 34.1 Hz ± 11.1 Hz; p value < 0.05), which also resolved by day three (Figure 2D); however, unlike mechanical thresholds in AM/APMs, heat thresholds in the APM afferents were significantly reduced at one (38.6°C ± 2.3°C; p value < 0.05) and three (34.8°C ± 0.5°C; p value < 0.05) days after P7 inflammation compared to baseline (48.4°C ± 2.7°C). Heat thresholds returned to naive levels (42.4 ± 4.1°C) by day seven (Figure 2B). These results were consistent regardless if we compared the inflamed time points to only naïve P7 AM/APMs (Figure 2) or the combined data obtained from mice < P14 (not shown). However, due to the fact that only two heat sensitive APM neurons were detected seven days post inflammation at P7, we also combined all of the data generated from mice with cutaneous inflammation at P7 and found similar results in that hairy skin inflammation at P7 causes a reduction in the heat thresholds (47.1°C ± 1.7°C, n = 5 vs 38.6°C ± 1.6°C, n = 11; p value < 0.05) and enhances firing to heat stimuli (3.5 Hz ± 1.7 Hz, n = 5 vs. 24 Hz ± 8.2 Hz, n = 11; p value < 0.05) in these afferent subtypes.

#### ***P7 Inflamed C-fibers***

In contrast to results obtained from A-fiber neurons, no changes in mechanical (Naïve: 21.7 mN ± 14.2 mN; 1d: 20.3 mN ± 15.1 mN; 3d: 33.8 mN ± 22.3 mN; 7d: 28.6 mN ± 18.3 mN; p value < 0.96) or heat (Naïve: 47.2°C ± 2.5°C; 1d: 41.4°C ± 2.4°C; 3d: 41.0°C ± 1.1°C; 7d: 43.4°C ± 1.1°C; p value < 0.16) thresholds, or firing (Mechanical: Naïve: 52.2 Hz ± 27.7 Hz; 1d: 55.0 Hz ± 15.5 Hz; 3d: 43.3 Hz ± 11.5 Hz; 7d: 28.5 Hz ± 5.4 Hz; p value < 0.24; Heat: Naïve: 5.0 Hz ± 2.7 Hz; 1d: 10.1 Hz ± 4.6 Hz; 3d: 7.0 Hz ± 2.2 Hz; 7d: 9.4 Hz ± 2.3 Hz; p value < 0.74) to these peripheral stimuli were observed in the CPM neurons (Mechanical responses: Naive: n = 3; 1d: n = 6; 3d: n = 10; 7d: n = 19; Heat responses: Naive: n = 3; 1d: n = 7; 3d: n = 12; 7d: n = 15) at any time point after P7 inflammation of the hairy skin.

We also did not observe a statistically significant change in mechanical thresholds (Naïve: 21.0 mN ± 9.7 mN; 1d: 34.5 mN ± 13.5 mN; 3d: 66.0 mN ± 8.7 mN; 7d: 38.8 mN ± 17.0 mN; p value < 0.1) in the CM fibers (Naive: n = 11; 1d: n = 6; 3d: n = 12; 7d: n = 9) after P7 inflammation. Relative to naive mice (74.6 Hz ± 8.0 Hz), although no differences were found one day after carrageenan injection (97.4 Hz ± 12.0 Hz), we did find three (27.5 Hz ± 7.6 Hz) and seven (34.9 Hz ± 11.3 Hz) days after inflammation that CM fibers had lower firing to mechanical stimulation of the hairy skin (p value < 0.05). However, this is no different than the previously observed reduction in firing in these afferents during normal development using age-matched comparisons (see above). There was a statistically significant increase in mechanical firing one day after inflammation however, only when comparing results to time matched naives at P8 (Naïve: 50.6 Hz ± 9.8 Hz (n = 15); 1d inflamed: 97.4 Hz ± 12.0 Hz; p value < 0.05). Finally, we also found no differences in the response properties of CH neurons at any time point after P7 inflammation; however, low sampling prevents us from making firm statements about this result since few CH fibers were recorded and insufficient physiological data was obtained from this subtype at the various time points post P7 inflammation (not shown).

#### ***P14 Inflamed A-fibers***

To then determine if a different pattern of afferent sensitization occurred after inflammation in the more developed animals, we performed *ex vivo* recording in mice with hairy skin inflammation initiated at P14. Paw edema was also consistent to that observed during P7 inflammation (above). At P14 the paw volume ratio between ipsilateral and contralateral hindpaws was 1.7 ± 0.1 at 1d, 1.5 ± 0.1 at 3d and 1.5 ± 0.1 at 7d. Interestingly, we found no differences in the response properties of the AM/APM neurons at any time point after P14 inflammation. No changes in mechanical thresholds (Naïve: 38.3 mN ± 30.9 mN; 1d: 11.3 mN ± 6.0 mN; 3d: 29.0 mN ± 13.3 mN; 60.0 mN ± 18.6 mN; p value < 0.19) or firing (Naïve: 75.4 Hz ± 69.6 Hz; 1d: 94.5 Hz ± 19.7 Hz; 3d: 76.1 Hz ± 14.5 Hz; 7d: 58.4 Hz ± 16.5 Hz; p value < 0.8) to mechanical deformation of the skin were detected (Naive: n = 3; 1d: n = 6; 3d: n = 8; 7d: n = 5). We also observed no changes in heat thresholds (Naïve: 42.6°C ± 5.3°C; 1d: 43.1°C ± 2.7°C; 3d: 39.9°C ± 3.1°C; 7d: 46.8°C ± 0°C; p value < 0.76), or firing (Naïve: 1.5 Hz ± 1.3 Hz; 1d: 0.6 Hz ± 0.4 Hz; 3d: 17.9Hz ± 13.7Hz; 7d: 1.3 Hz ± 0 Hz; p value < 0.63) to heat stimuli in these afferents subtypes (Naive: n = 2; 1d: n = 2; 3d: n = 3; 7d: n = 1).

These same results were obtained if we compared this data to the combined naïve AM/APMs from ages ≥ P14 (not shown). However, since we acquired a small number of heat sensitive afferents after P14 inflammation at the various time points, we also combined all data obtained from mice with cutaneous inflammation induced at P14. We again found that no differences were observed in heat thresholds (43.4°C ± 2.3°C, n = 4 vs 42.1°C ± 1.9°C, n = 6; p value < 0.68) or firing to heat stimuli (4.0 Hz ± 2.7 Hz, n = 3 vs. 9.3 Hz ± 7.2 Hz, n = 6; p value < 0.63) after P14 inflammation.

#### ***P14 Inflamed C-fibers***

**Figure 3:**
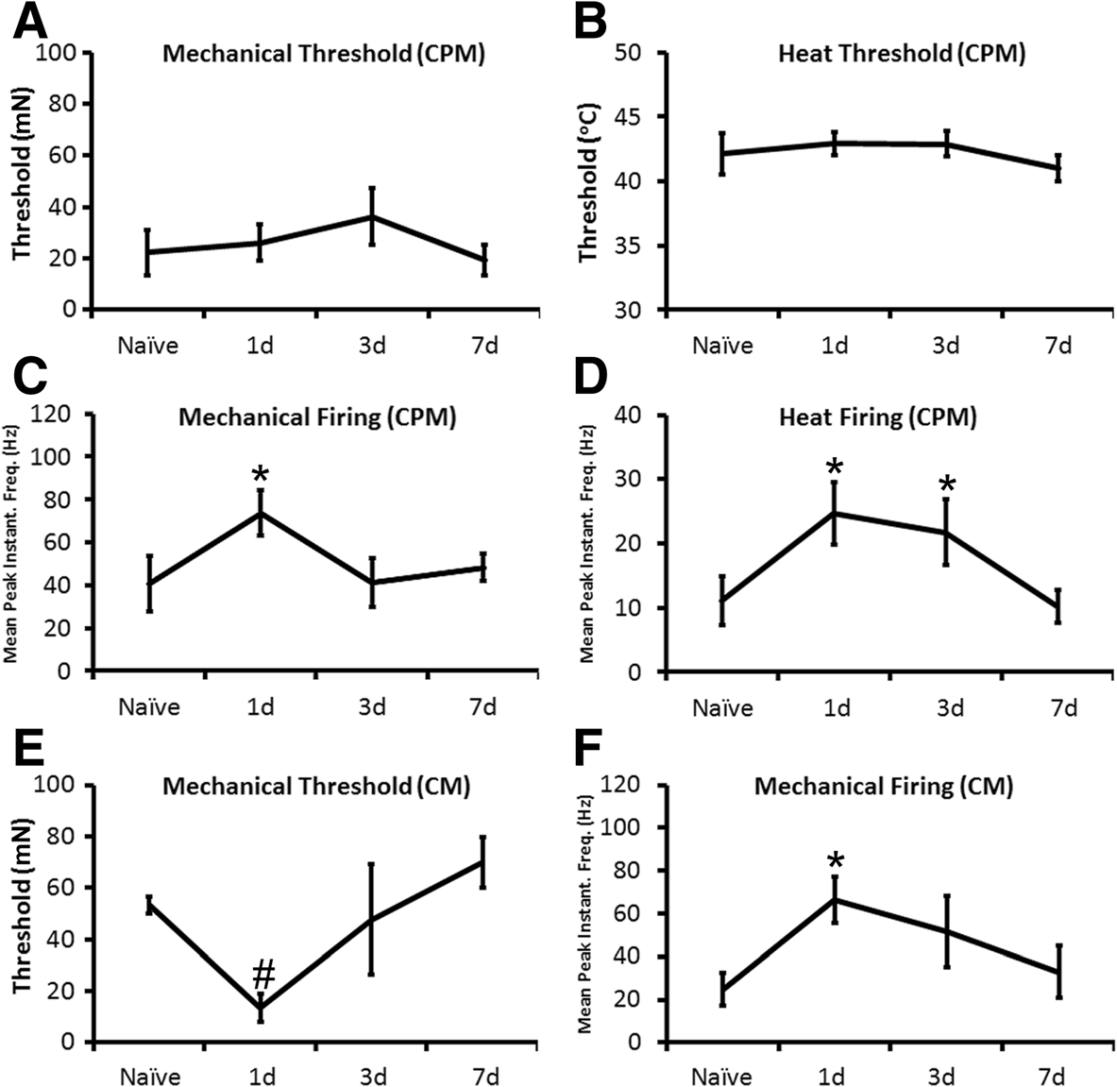
**Response properties of mechanically sensitive, thermally insensitive (CM) and polymodal (CPM) C-fibers after P14 inflammation. A**: No changes were observed in the mechanical thresholds of CPM neurons at any time point after inflammation of the hairy skin at P14. **B**: No differences in heat threshold in the CPM fibers were detected after P14 inflammation. **C**: However, there was an increase in the mean peak instantaneous frequencies to mechanical stimulation of the skin in the CPM neurons one day after P14 inflammation. This effect was transient as firing returned to naïve levels by the three day time point. **D**: Similar results were found in terms of the mean peak instantaneous frequencies to heat stimuli in the CPM fibers. However, the enhanced firing to heat was maintained at the three day time point and resolved by day seven. **E**: Although there was not a statistically significant reduction in mechanical thresholds in the CM neurons, there was a trend towards reduced mechanical thresholds in these afferents one day after injection of carrageenan into the hairy skin at P14. **F**: The CM fibers however did display increased mean peak instantaneous frequencies to mechanical stimulation of the skin one day after P14 inflammation. *p value <0.05; ^#^p value < 0.1, mean ± SEM; One-way ANOVA with Tukey’s post hoc test for thresholds and Kruskal-Wallis with Dunn’s post hoc tests for mean peak instantaneous frequencies. See text for animal and afferent numbers.

Instead of detecting changes in A-fibers after P14 inflammation of the hairy skin, we found significantly increased mean peak instantaneous frequencies to both mechanical (40.7 Hz ± 12.7 Hz; vs. 73.7 Hz ± 10.5 Hz; p value < 0.05) and heat (11.2 Hz ± 3.8 Hz; vs. 24.7 Hz ± 4.9 Hz; p value < 0.05) stimuli in the CPM fibers (Mechanical responders: Naive: n = 11; 1d: n = 23; 3d: n = 11; 7d: n = 15; Heat responders: Naive: n = 14; 1d: n = 23; 3d: n = 18; 7d: n = 17). Responsiveness to heat stimuli was also elevated at the 3d time point in these afferents (21.8 Hz ± 5.1 Hz; p value < 0.05). No changes however were found in mechanical (Naïve: 22.3 mN ± 8.7 mN; 1d: 26.1 mN ± 7.1 mN; 3d: 36.3 mN ± 10.9 mN; 7d: 19.3 mN ± 5.9 mN; p value < 0.55) or heat (Naïve: 42.1°C ± 1.6°C; 1d: 42.9°C ± 0.9°C; 3d: 42.9°C ± 0.9°C; 7d: 41.0°C ± 1.1°C; p value < 0.53) thresholds in these afferent subtypes (Figure 3A-D).In addition to changes observed in the CPM population, we also observed changes in the CM fibers (Naive: n = 8; 1d: n = 8; 3d: n = 5; 7d: n = 6). One day after inflammation at P14, we found reduced mechanical thresholds (Naïve: 53.3 mN ± 3.3 mN vs. 1d: 13.3 mN ± 5.4 mN); however, this was not statistically significant overall (p value < 0.1). Nevertheless, we did find increased firing (Naïve: 25.0 Hz ± 7.6 Hz vs. 1d: 66.6 Hz ± 10.7 Hz; p value < 0.05) to mechanical stimuli in the CMs (Figure 3E, F). Firing to mechanical stimuli in the CM population was also elevated from naïve levels at 3d (51.9 Hz ± 16.5 Hz; p value < 0.21) post inflammation, but this was not found to be statistically significant. There were also no significant changes in firing compared to baseline at day seven (33.0 Hz ± 12.2 Hz; p value < 0.52) in these cell types.

### Age-specific changes in the prevalence of cutaneous afferents during neonatal inflammation

**Figure 4:**
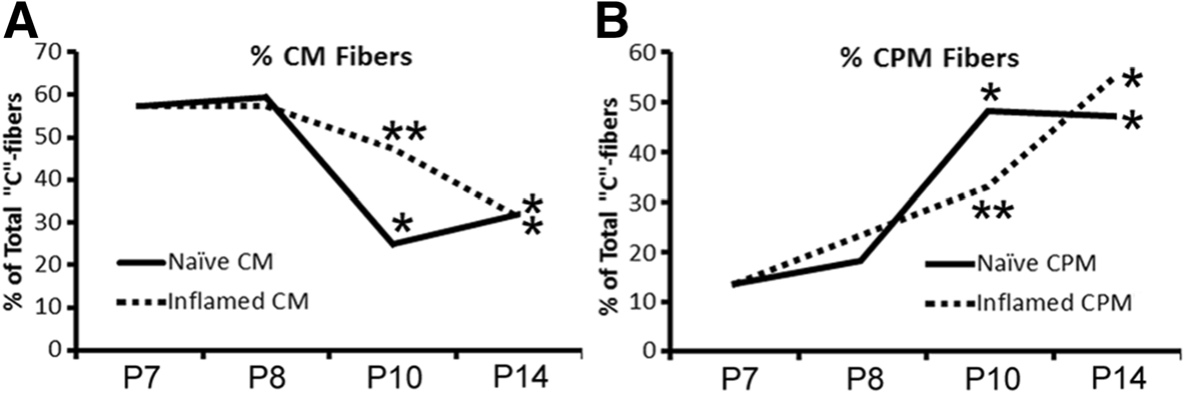
**Prevalence of mechanically sensitive, thermally insensitive (CM) and polymodal (CPM) “C”-fibers after inflammation at P7. A**: The reduction in the percentage of total CM fibers at P10 in naïve mice was delayed by cutaneous inflammation at P7. The percentage of CM neurons detected three days after P7 inflammation (P10) was not found to be different than the percentage of these cells in naives at P7 or at one day post carrageenan injection (P8). The numbers of CM neurons detected at the three day time point was significantly higher than that detected in time matched naives at P10. By seven days post inflammation (P14), there was no difference in CM fiber prevalence between naives and inflamed mice. **B**: The normal increase in CPM neuron prevalence at P10 in naïve mice was also delayed by inflammation at P7. The percentage of CPM neurons detected three days after P7 inflammation of the hairy skin was significantly lower than the numbers of CPM neurons found in time matched naives (P10). The increase in CPM neuron prevalence was resolved however by the seven day time point after inflammation (P14). *p value < 0.05 vs P7 and P8 (1d post inflammation); **p value < 0.05 vs time matched naïve controls (P10), *χ*^2^. See text for animal and afferent totals.

In addition to changes in response properties, we also observed changes in the numbers of specific afferent types recorded; however, changes in the percentage of particular afferents were only found after P7 inflammation. When we analyzed the percentages of total C-fibers, we observed significant changes in the numbers of both CM and CPM neurons after P7 hairy skin inflammation compared to normal development. While naïve mice displayed a shift in the frequency of CM and CPM neurons between P8 and P10 (Figure 1), inflammation of the hairy skin caused a delay in this switch. That is to say, three days after P7 inflammation (P10), there were no differences in CM (47%, n = 17/36) or CPM (33%, n = 12/36) neuron prevalence relative to P7 naïves (CM: 57%, n = 17/30; CPM: 13%, n = 4/30) or one day after inflammation (CM: 57%, n = 17/30; CPM: 23%, n = 7/30) in contrast to the shift seen in uninjured mice during this time period. However, by seven days, both CM (31%, n = 13/42) and CPM (55%, n = 23/42) fiber numbers were similar to age-matched controls (Figure 4). CH neuron prevalence was not altered by P7 inflammation (Naïve: 13%, n = 4/30; 1d: 13%, n = 4/30; 3d: 6%, n = 2/36; 7d: 5%, n = 2/42; p value < 0.41).

In addition, when we analyzed AM/APM fiber numbers, although no statistical differences were found in the numbers of AM fibers 1–7 days after inflammation induced at P7 (Naïve: 28%, n = 5/18; 1d: 44%, n = 11/25; 3d: 57%, n = 8/14; 7d: 36%, n = 5/14; p value < 0.38), we did observe a transient increase (36%, n = 9/25; p value < 0.044) in the numbers of the heat sensitive APM neurons one day after inflammation relative to P7 naives (17%, n = 3/18) that resolved by day three (13%, n = 2/14) and was maintained at day seven (21%, n = 3/14; not shown). However, unlike after P7 inflammation, P14 inflammation did not alter the percent of total fibers analyzed at any time point tested (AM: Naïve: 23%, n = 3/13; 1d: 21%, n = 3/14; 3d: 36%, n = 5/14; 7d: 50%, n = 3/6; p value < 0.54; APM: Naïve: 15%, n = 2/13; 1d: 21%, n = 3/14; 3d: 29%, n = 4/14; 7d: 33%, n = 2/6; p value < 0.79; CPM: Naïve: 47%, n = 18/38; 1d: 59%, n = 27/46; 3d: 56%, n = 20/36; 7d: 46%, n = 22/48; p value < 0.56; CM: Naïve: 32%, n = 12/38; 1d: 24%, n = 11/46; 3d: 31%, n = 11/36; 7d: 21%, n = 10/48; p value < 0.97; CH: Naïve: 11%, n = 4/38; 1d: 4%, n = 2/46; 3d: 3%, n = 1/36; 7d: 13%, n = 6/48; p value < 0.27).

### Neurochemical identities of cutaneous afferents during neonatal inflammation

#### ***Naïve sensory fibers***

**Figure 5:**
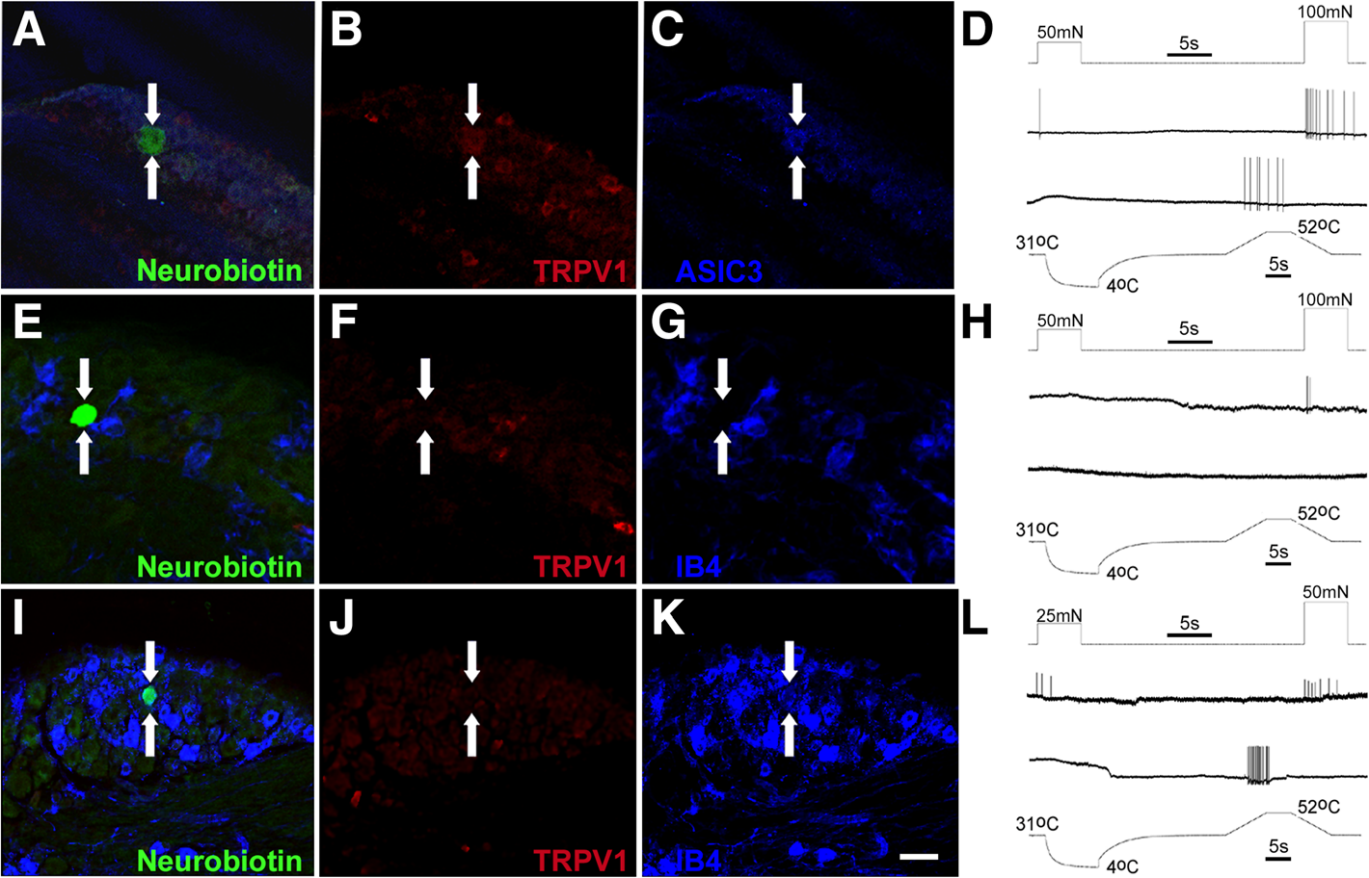
**Intracellularly stained and physiologically characterized sensory neurons in naïve mice from the *ex vivo *preparation.** A neurobiotin stained “A”-fiber neuron (arrows) from a P7 mouse **(A)** that responded to mechanical stimuli and heat stimuli **(D)** was found to be immunoreactive for both TRPV1 **(B; red)** and ASIC3 **(C; blue).** One “C”-fiber neuron (arrows) recovered from a P8 mouse that was intracellularly stained with neurobiotin **(E)** and responded to mechanical deformation of the skin but not heat stimuli **(H)** was found to be immunonegative for TRPV1 **(F; red)** and did not bind isolectin B4 **(IB4; G; blue)**. A polymodal **(L)** “C”-fiber neuron intracellularly stained with neurobiotin **(I)** and recovered from a P21 naïve *ex vivo* preparation (arrows) was found to be TRPV1 immunonegative **(J; red),** but did bind IB4 **(K; blue)**. Scale bar for images: 40 μm.

To then assess whether there could be corresponding changes in afferent phenotype in addition to the changes in response properties observed after inflammation at either P7 or P14, we intracellularly stained 36 cells from the various *ex vivo* preparations with neurobiotin and processed the DRGs containing single cells for IB4 binding, TRPV1 immunostaining or ASIC3 immunoreactivity. These markers were chosen since it has been previously shown that these molecules often mark specific sensory neuron subpopulations in adults [32,33,38,39] and the onset of IB4 staining is thought to occur around the same developmental time period being analyzed in this study [24]. In naïve mice at postnatal days seven and eight (prior to the functional changes in sensory afferents), we found that no CM (0 of 5) or CPM (0 of 1) fibers were immunopositive for the heat transducing channel TRPV1, nor did any of these afferent subtypes bind IB4 (Figure 5; Table 2).

**Table 2:** **Immunostaining results from intracellularly stained sensory neurons recovered from the *ex vivo *** **preparation**

***Naive***	**Postnatal Day 7/8**			**Postnatal Day 10≤**		
**Cell Type**	**TRPV1**	**IB4**	**V1+/IB4+**	**TRPV1**	**IB4**	**V1+/IB4+**
CM	0/5	0/5	0/5	0/1	0/1	0/1
CPM	0/1	0/1	0/1	0/6	4/8	0/6
***Inflammation***	**P7 Inflammation**			**P14 Inflammation**		
**Cell type**	**TRPV1**	**IB4**	**V1+/IB4+**	**TRPV1**	**IB4**	**V1+/IB4+**
CM	0/2	0/2	0/2	1/1	0/1	0/1
CPM	4/8	4/9	2/8	3/7	1/7	1/7

Both naïve and peripherally inflamed mice are represented in the table.

After P10, the one CM fiber that was neurochemically characterized still was not found to bind IB4 or stain positively for TRPV1. Although, the CPM fibers were often (4 of 8) found to bind IB4, they still were not immunoreactive for TRPV1 (Figure 5) at this age (≥P10), consistent with results obtained from adult mice [32,33,38]. In addition, we intracellularly filled one APM fiber at P7, two mechano-cold (MC) fibers (one with “A”-fiber CVs and one with “C”-fiber CVs) at P10 and one CH neuron at P14. The APM was found to be immunopositive for both TRPV1 and ASIC3 (Figure 5). The CH neuron was found to be TRPV1 positive and IB4 negative, and the two MC fibers were both found to be immunonegative for TRPV1 and did not bind IB4 (not shown) similar to adult animals (e.g. [32,33,38]).

#### ***Inflamed sensory fibers***

**Figure 6:**
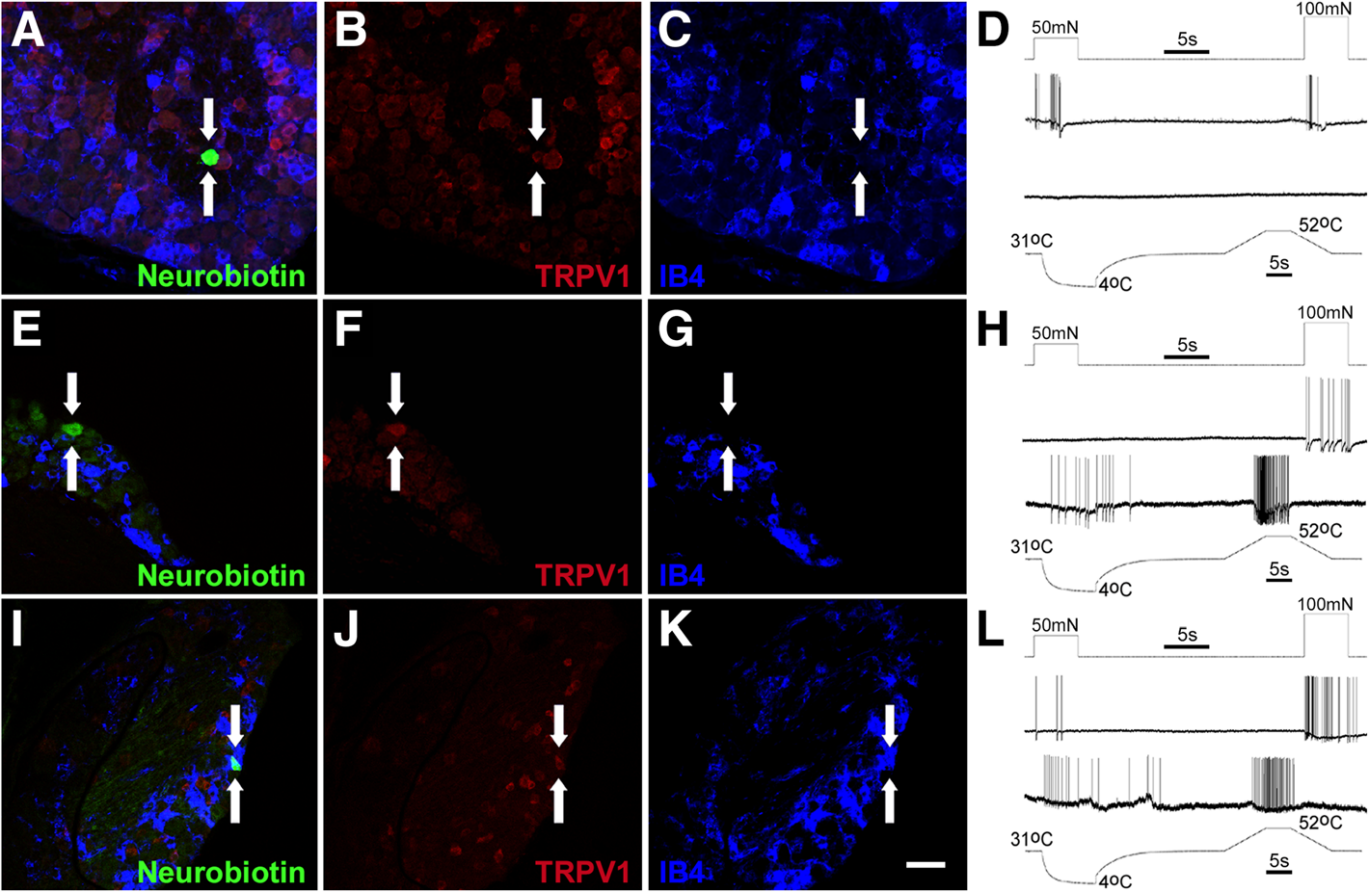
**Intracellularly stained and physiologically characterized afferents in mice with inflammation recovered after *ex vivo *recording.** A neurobiotin (NB) stained **(A)** “C”-fiber neuron (arrows) that was recovered from a mouse seven days after P14 cutaneous inflammation was found to be mechanically sensitive but thermally insensitive **(D)**, and was found to contain TRPV1 protein **(B; red)** but did not bind isolectin B4 **(IB4; C; blue)**. The TRPV1 staining in this particular neuron did not appear to be membrane bound as the labeling pattern for TRPV1 was within the staining pattern for NB **(A)**. One polymodal **(H)** “C”-fiber (arrows) that was filled with NB **(E)** was found to contain TRPV1 **(F; red)** but did not bind IB4 **(G; blue)**. This cell was recovered from a mouse with cutaneous inflammation induced at P14 (three day time point). Finally, another polymodal “C”-fiber **(L; arrows)** that was intracellularly filled with NB **(I)** was found to contain both TRPV1 **(J; red)** and bound IB4 **(K; blue)**. This latter cell was obtained from a mouse with inflammation at P7 at the one day time point. Scale bar for images: 40 μm.

After inflammation at P7, CM fibers were still found to be negative for both TRPV1 and IB4 binding (Table 2); however, the few CPM fibers that were detected did show interesting neurochemical phenotypes. In fact, after inflammation at P7, we detected IB4 binding in these afferent subtypes in addition to select cells with TRPV1 staining. Some CPM neurons were also found to be positive for both IB4 and TRPV1 (Figure 6; Table 2). These results were not found to be specific to any time point after inflammation as TRPV1 or IB4 positive cells were found at 1d (prior to the functional changes in “C”-fiber afferents), and 3d or 7d (after the observed functional changes) post P7 inflammation.

After P14 inflammation, results were similar to P7 inflammation for the CPM neurons as some afferents (3 of 7) were found to be immunoreactive for TRPV1, unlike what was found in naïve mice (Figures 5, 6; Table 2). CPM neurons were still found to bind IB4 after P14 inflammation; however, fewer cells (1 of 7) were found to be positive for this marker compared to naives. This same IB4 positive CPM was also found to be immunopositive for TRPV1 (Figure 6I-L). Interestingly, the one CM fiber that was intracellularly filled with neurobiotin and immunocytochemically processed after P14 inflammation was found to be IB4 negative, but TRPV1 positive (Figure 6A-D; Table 2). However, the TRPV1 staining appeared to be intracellular and not at the cell membrane in this CM fiber. Additionally, we stained one APM fiber and one CH fiber after P14 inflammation. Both of these cells were found to be TRPV1 immunoreactive but not IB4 binding (not shown). In summary, IB4 binding appears to be detected in specific sensory neurons subtypes (CPM) after P10, but inflammation either before or after this time point may potentially promote TRPV1 staining in these cell types.

### Cutaneous inflammation age-dependently alters gene expression in the DRGs

In order to determine potential mechanisms for the observed changes in afferent function and phenotype after P7 or P14 inflammation, we then performed realtime PCR analysis of neonatal DRGs one and three days after P7 or P14 inflammation and compared expression levels to age-matched naïve DRGs (Table 3; n = 3-5 per condition). We first analyzed the expression of neurotrophic factor and cytokine receptors in the DRGs. We found a reduction in the NGF receptor trkA (P7: 1d: -46 ± 22%; 3d: -99 ± 30%*; P14: 1d: -46 ± 17%; 3d: -92 ± 20%*; *p value < 0.05) and the artemin neurotrophic factor co-receptor GFRα3 (P7: 1d: -47 ± 14%*; 3d: -99 ± 9%*; P14: 1d: -32 ± 12%; 3d: -91 ± 11%*; *p value < 0.05). We also observed a reduction in the GDNF co-receptor GFRα1, but this was only found 3d after P7 inflammation (-69 ± 35%; p value <0.05) and not after P14 inflammation. Increased expression however was found in the type 1 receptor for insulin like growth factor 1 (IGFr1; P7: 1d: 96 ± 36%*; 3d: 754 ± 34%*; P14: 1d: 169 ± 19%*; 3d: 831 ± 31%*; *p value < 0.05), and the receptor for the cytokine interleukin 1β (IL1r1; P7: 1d: 257 ± 31%*; 3d: 268 ± 15%*; P14: 1d: 50 ± 22%; 3d: 387 ± 12%*; *p value < 0.05) after both P7 and P14 inflammation.

**Table 3:** Percent changes in mRNA expression in the L2/L3 DRGs after inflammation

**Gene**	**P7 Inflammation (1d)**	**P7 Inflammation (3d)**	**P14 Inflammation (1d)**	**P14 Inflammation (3d)**
trkA	-46 ± 22^#^	-99 ± 30*	-46 ± 17	-92 ± 20*
GFRα1	-29 ± 27	-69 ± 35*	-1 ± 9	18 ± 10
GFRα3	-47 ± 17*	-99 ± 9*	-32 ± 12	-91 ± 11*
IGFr1	98 ± 36*	754 ± 34*	169 ± 19*	831 ± 31*
IL1r1	257 ± 31*	268 ± 15*	50 ± 22	387 ± 12*
ASIC1	18 ± 25	-15 ± 31	-18 ± 45	-11 ± 9
ASIC3	115 ± 24*	16 ± 34	-19 ± 60	-28 ± 7
TRPA1	143 ± 37*	-38 ± 14	83 ± 29*	-7 ± 12
TRPC3	-25 ± 23	-30 ± 7	84 ± 29*	-57 ± 39
TRPM3	52 ± 11*	-30 ± 20	4 ± 18	-36 ± 22
TRPM6	174 ± 33*	-20 ± 22	159 ± 29*	-47 ± 51
TRPV1	1 ± 28	52 ± 35	69 ± 10*	10 ± 76
P2Y1	107 ± 22*	-23 ± 20	-31 ± 27	-50 ± 40
P2X3	-16 ± 15	40 ± 26	29 ± 15	21 ± 5
Piezo2	-14 ± 18	60 ± 20	58 ± 11*	87 ± 9*

Data presented are percent changes in expression from DRGs with cutaneous inflammation induced at P7 or P14 by hairy skin injection of carrageenan compared to age-matched naives. *p value < 0.05; #p value < 0.1. Mean ± SEM; One-way ANOVA with Tukey’s post hoc test.

Although the irritant receptor TRPA1 was found to be upregulated 1d after both P7 (143 ± 37%; p value < 0.05) and P14 (83 ± 29%; p value < 0.05) inflammation, as was the Mg^2+^ channel, TRPM6 (P7: 174 ± 33%; P14: 159 ± 29%; p values < 0.05), most receptors/channels that have been classically linked to sensory transduction were differentially altered in the DRGs after inflammation at P7 compared to P14. We found after P7 inflammation that ASIC3 (a receptor linked to pH sensitivity and mechanotransduction) was upregulated 1d after peripheral injury (115 ± 24%; p value < 0.05). In addition, we also detected a significant upregulation of the heat channel TRPM3 (52 ± 11%; p value < 0.05) and the heat threshold modulator and purinergic receptor, P2Y1 (107 ± 22%; p value < 0.05) 1d after P7 inflammation. Conversely, after P14 inflammation, we observed a unique set of changes in gene expression compared to P7. Specifically, we found a significant upregulation of the excitability regulator TRPC3 (84 ± 29%; p value < 0.05) and the heat transducing channel TRPV1 (69 ± 10%; p value < 0.05) 1d after P14 inflammation of the hairy skin. In addition, we also found that the mechanotransducer Piezo2 was upregulated in the DRGs after P14 inflammation at both 1d (58 ± 11%; p value < 0.05) and 3d (87 ± 9%; p value < 0.05). No changes in expression were observed in ASIC1 or P2X3 after inflammation at either P7 or P14 (Table 3).

**Figure 7:**
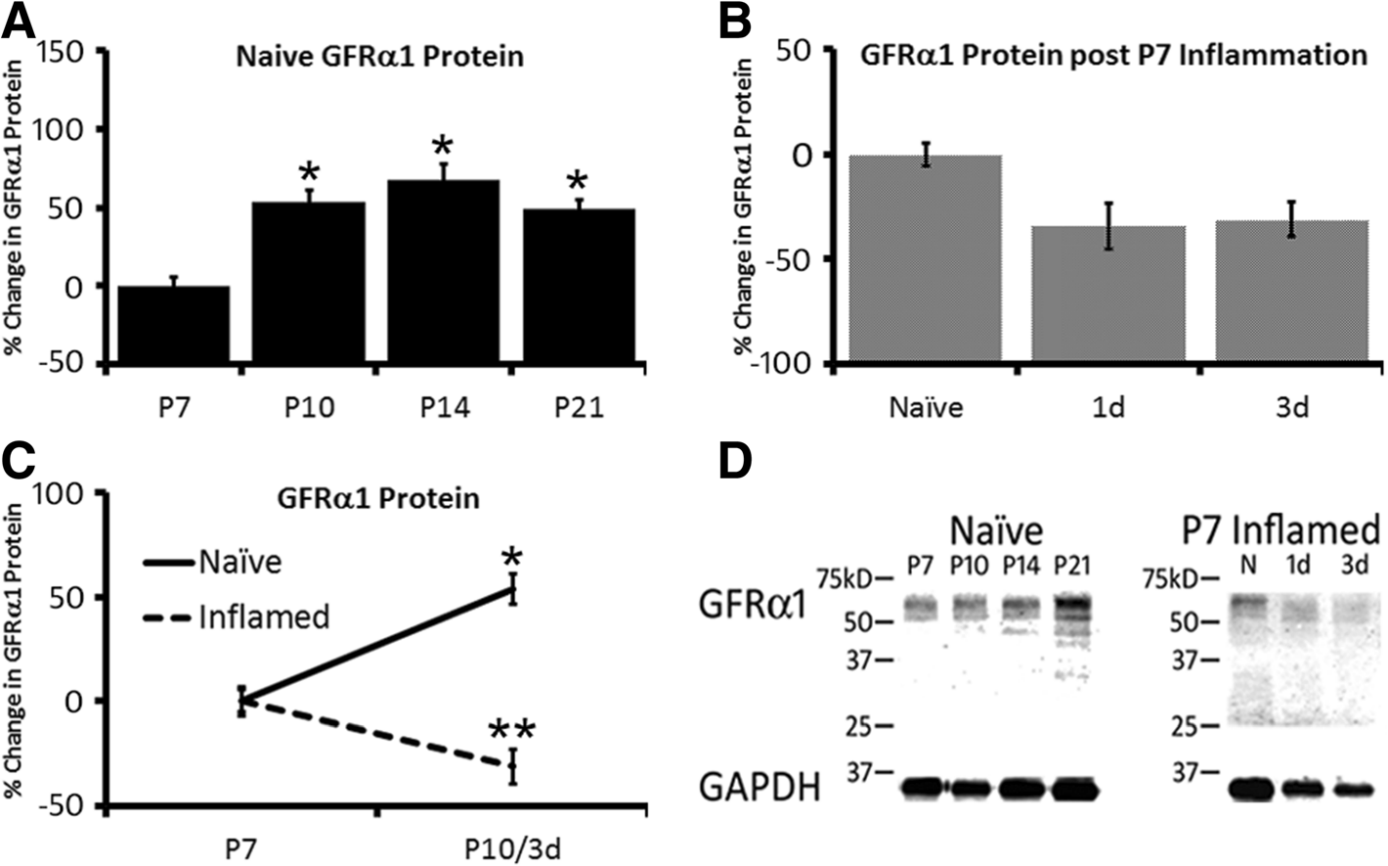
**Western blot analysis of whole DRGs in naïve and P7 inflamed mice for GFRα1. A**: Quantification of GFRα1 protein in naïve L2/L3 DRGs from P7 through P21 revealed that levels of this co-receptor were significantly elevated beginning at P10 and remained elevated thereafter compared to P7 levels. **B**: However, after cutaneous inflammation at P7, GFRα1 protein did not display enhanced expression at one day or three days after inflammation; the age in which it normally increased in naïve ganglia (P10). In fact, there appeared to be a slight decrease in the mean expression of GFRα1 protein after P7 inflammation, but this did not reach statistical significance. **C**: Comparing levels of GFRα1 protein between P7 and P10 (which is also 3d post inflammation) in naïve and inflamed mice shows that inflammation of the hairy hindpaw skin blocked the normal increase in this receptor normally seen in naïve mice. **D**: Examples of western blots for GFRα1 protein in naïve mice from P7 through P21 or after P7 inflammation at 1d and 3d. Bands are detected around the predicted molecular weights for GFRα1 and GAPDH. *p value <0.05 vs P7 naives. Values for GFRα1 are normalized to GAPDH levels prior to quantification. Normalized mean ± SEM; One-way ANOVA with Tukey’s post hoc test. Presented as percent changes.

To confirm that our changes in mRNA expression were likely followed by similar upregulation of protein expression, we performed western blotting analysis on GFRα1 in naïve L2/L3 DRGs and in the L2/L3 DRGs of mice after P7 or P14 inflammation since changes in this particular receptor could be linked to the alterations in afferent prevalence (Figures 1, 4) detected during neonatal development [22,29] and after inflammation. As anticipated, we found that the average total GFRα1 protein was significantly upregulated in the DRGs beginning at P10 (Figure 7A, D) compared to the DRGs from P7 naïve mice (53 ± 7%; p value < 0.05), but after P7 inflammation (Figure 7B, D), the average total GFRα1 protein in the L2/L3 DRGs was slightly downregulated relative to P7 naïve DRGs at the 1d (-35 ± 11%) and 3d (-31 ± 8%) time points, although this was not statistically significant (p value < 0.1). When comparing the changes in naïve GFRα1 protein in the DRGs over time to time-matched inflamed DRGs, we found that peripheral inflammation appeared to block the normal upregulation of total GFRα1 protein expression at P10 (Figure 7C, D). No changes in GFRα1 protein were found after P14 inflammation similar to the pattern of mRNA expression (not shown).

## Discussion

**Figure 8:**
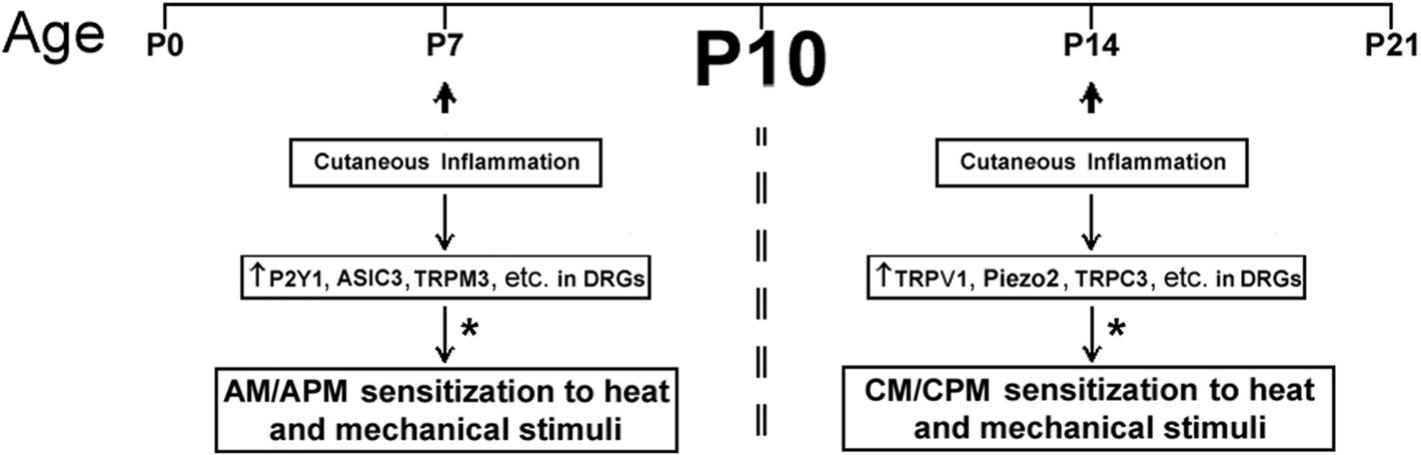
**Schematic representation of the age-dependent sensitization of cutaneous sensory neurons during developmental inflammation.** Peripheral injury (cutaneous inflammation) sustained prior to the observed critical period (P10; bold) of sensory neuron development (at P7; arrow) specifically results in sensitization of A-fibers (AM/APM; left). Inflammation of the skin after this time point however (at P14; arrow) only sensitizes mechanically sensitive, thermally insensitive (CM) and polymodal (CPM) C-fibers to peripheral stimuli (right). We hypothesize that these unique age-related changes in afferent responsiveness may be due to upregulation of specific genes in the DRGs at the different time points (*).

Reports have shown that neonates of all ages develop mechanical and thermal hyperalgesia after peripheral tissue injury (e.g. [6,40,41]). The current study has uncovered two novel findings in how sensory neurons may play a role in neonatal pain development. We have first shown that the prevalence of the CM and CPM afferent subtypes are changing between the first and second weeks of life. This coincides with the time period in which sensory neurons are vulnerable to neurotrophic factors in the periphery for their functional development [25-28] and are switching their neurotrophic factor sensitivity from NGF to GDNF [22,24]. Next, we have shown that the sensitization that occurs in specific sensory neuron populations is also different depending on the age at which peripheral injury was induced (Figure 8).

### Gene expression and sensory neuron development

It is a known phenomenon that specific sensory neurons that express trkA will down-regulate this receptor postnatally and increase expression of the ret tyrosine kinase receptor [22,24]. This changes the sensitivity of these sensory neurons from NGF to GDNF, which regulates their proper development. We have found that at the same time trkA is downregulated and the ret receptor and GDNF co-receptor GFRα1 is upregulated (Table 1; Figure 7), the prevalence of CM neurons is decreased with a corresponding increase in the CPMs (Figure 1). This may suggest that these mechanically sensitive afferents are gaining heat responsiveness at the time they become responsive to GDNF. In support of this notion, mice that overexpress GDNF in the skin have significantly more heat responsive C-fibers [29]. Thus the observed (Table 1, Figure 7) increase in GFRα1 expression in the DRGs may not only serve to support the normal maturation of sensory neurons [22], but also may play a role in the functional switch that occurs at the same time. In addition, mice that overexpress NGF in the skin also display increased numbers of heat sensitive C-fibers [42]. NGF is thought to regulate afferent function (e.g. [26-28]) and GDNF dependent maturation of sensory neurons [22]. Thus it would also be interesting to determine how NGF itself alters CM and CPM fiber frequency during this critical period in future experiments.

Changes in other receptors/channels in the DRGs may also play a role in altered function of individual subtypes. We observed a reduction in mechanical responsiveness in the CM population between P8 and P10. This altered response property correlated with our observed decrease in excitability regulator TRPC3 ([43]; Table 1). Thus alterations in functional phenotype may be driven by changes in neurotrophic factor responsiveness while the limited changes in response properties in individual afferents are likely due to changes in specific receptors/channels associated with sensory transduction.

### Age-dependent sensitization of cutaneous sensory neurons during inflammation

Although *individual* afferent response properties do not generally change during development ([19,21,30,44]; current report), we and others [30] have shown that the prevalence of afferent subtypes does change. Myelinated afferents appear to lose heat sensitivity during the first week of life [30], while “C”-fibers may gain heat sensitivity during the second week (Figure 1). Thus if peripheral injury is experienced at the appropriate time, unique changes in primary afferents could generate neonatal pain in an age-related manner.

Here we examined whether peripheral inflammation immediately prior to our observed functional switch in afferent prevalence (P7) would produce a different set of changes in peripheral response properties compared to inflammation after the switch (P14). We found that the “A”-fiber nociceptors showed reduced thresholds to heat stimuli in addition to enhanced firing to both mechanical and heat stimulation of the skin after P7 inflammation (Figure 2). Widespread changes in afferent response properties were not observed in the “C”-fibers (not shown). This is different to that observed during P14 inflammation where the plasticity that is observed in sensory neurons occurs specifically in the “C”-fibers and not the “A”-fibers. Both CM and CPM neurons at this time showed increased responsiveness to mechanical stimuli and the CPM neurons also displayed enhanced heat responses (Figure 3). Thus even though individual afferents appear to be fully mature in terms of their peripheral response properties from as early as P0 in naïve animals (e.g. [19,21,30]), how these developing afferents respond to injury appears to be quite different depending on the stage of development that injury is sustained (Figure 8). Therefore, acute pain may be generated in neonates (e.g. [6,40,41]) through these unique types of alterations in afferents.

The mechanisms of how these changes may occur in sensory neurons after developmental inflammation may also be due to unique changes in gene expression in the DRGs since reports in adult animals have shown that *dynamic* changes in DRG gene expression are critically linked to altered sensory function after peripheral injuries (e.g. [38,45,46]). After P7 inflammation, we found a unique increase in the pH sensor and mechanical modulator ASIC3 [47,48], the heat transducer TRPM3 [49] and the heat threshold regulator P2Y1 [45,50] in the DRGs (Table 3). These specific changes in gene expression correlate well with the observed changes in mechanical and heat responsiveness in addition to the reduction in heat thresholds in “A”-fiber neurons, respectively (Figure 2). Conversely, after P14 inflammation, we do not observe changes in these receptors, but we do observe an increase in the excitability regulator TRPC3 [43], the mechanotransducer piezo2 [51,52] and the heat channel TRPV1 [53,54] in the DRGs (Table 3). This correlates with the observed changes in mechanical responsiveness in the CPM and CM neurons and the enhanced responses to heat stimuli in the CPMs, respectively (Figure 3). Thus, not only are there unique subsets of afferents that sensitize after developmental inflammation depending upon the age at which injury is induced, but the changes in receptors/channels that may be mediating the altered peripheral responsiveness may also be age-dependent (Figure 8). Of course, we recognize the limitations of whole DRG analyses and acknowledge that a specific analysis of individual afferent function after developmental inflammation in the context of particular receptor inhibition will be necessary to clearly determine mechanisms.

Additionally, we observed changes in neurochemical identity in specific afferent types irrespective of postnatal age. Similar to previous reports in adult mice after peripheral injury [32,45,55] we found CPM neurons that expressed the TRPV1 channel after inflammation at both P7 and P14, which is not normally observed in naïve CPMs (Table 2; Figures 5, 6). If a similar phenomenon occurs in neonates as in adults, then the TRPV1 immunopositive CH neurons (not shown) may be gaining mechanical sensitivity, placing them in the CPM subcategory [32,45]. This was thought to be due to the fact that peripheral injury can upregulate IB4 binding in sensory neurons in adults [32]. In support of this idea in neonates, peripheral injuries can also induce IB4 staining in DRGs during early development [13]. However, a more detailed analysis of the neurochemical identities of single sensory neurons is necessary to confirm this notion.

## Conclusions/significance

Treatment for pain in children is often similar to that of adults; however, side effects from pharmacological agents such as opioids can be more harmful in children [56-58]. Thus development of more suitable pharmacotherapies for pediatric pain has been of great interest. To this end, key research in the developing spinal cord has been performed to determine how noxious information from the periphery is processed leading to altered acute and long-term pain perception (e.g. [9,59-64]). A central finding of these studies is that early neonatal injury accelerates dorsal horn circuit development likely via altered “A”-fiber inputs and erroneous “C”-fiber regulation of developing glycerinergic inhibition [64-68]. Our data presented here supports this finding in that we show enhanced responsiveness of the “A”-fiber nociceptors to mechanical and thermal stimuli after P7 inflammation (Figure 2) and a delay in the normal development of CM and CPM neurons (Figure 4). This latter finding may be due to reduced GFRα1 expression in the DRGs at this time after inflammation (Figure 7; Tables 1 and 3). Together, this may be a reason why early neonatal injury specifically causes long-term alterations of nociception and physiological function compared to peripheral injuries that occur later in life [9,59,60,62,63]. Peripheral injuries during this early time point in development appear to be altering sensory function which in turn incorrectly facilitates the development of spinal dorsal horn circuits and this may lead to subsequent alterations in supraspinal pathways and thus long-term alterations in nociception. Peripheral injury after the observed critical period of sensory development when peripheral fibers have fully matured could be one reason there are not long term affects seen when injuries are sustained later in development. This is a potentially important consideration for newborn infants or pre-term babies that undergo painful procedures or experience peripheral injuries [69,70].

Therefore a major consideration for pediatric pain management is the age in which peripheral injury is sustained. We have shown that the plasticity that occurs in individual sensory neurons and the upregulation of specific receptors/channels in the DRGs that may be mediating the changes in afferents is age-dependent during development and different than that observed in adults ([1,32,45]; current report). Thus the animal’s age, the afferents potentially mediating the pain and the receptors within those afferents are critical factors when formulating proper pain therapies for neonates.

## Methods

### Animals

Swiss Webster mice from postnatal day 0 through postnatal day 21 were used in these studies. All animals were housed with the mother, which was provided food and water *ad libitum* and maintained on a 12 hr light/dark cycle. All procedures were approved by the Institutional Animal Care and Use Committee at Cincinnati Children’s Hospital Medical Center, under AAALAC approved practices.

### Injection of carrageenan

All mice were anesthetized under 3% isofluorane during cutaneous injections. Using a syringe with a 30 g needle, 3-14 μL of 3% carrageenan (in 0.9% NaCl) was injected into the right hairy hindpaw skin. We used 1 μL/g body weight as a guide for these injections [64]. Measurements of paw edema (paw volume) using calipers on the ipsilateral hindpaw relative to the contralateral hindpaw at 1–7 days after injection ensured that a relatively similar degree of inflammation was achieved and maintained at the various developmental time points. Although this methodology does not specifically allow for conclusions to be drawn about the inflammatory process itself or the developmental regulation of peripheral inflammation, it does serve as a general guide to ensure we were not differentially inflaming the skin of mice at different ages/weights. Injections began at the ankle and the needle was slowly retracted as the agent was expelled from the syringe. A cotton swab was used after injections to prevent leakage of carrageenan. All electrophysiological, neurochemical or molecular analyses were performed 1d, 3d and 7d after injection of carrageenan and compared to naïve mice between P0 and P21 or with each other.

### Ex vivo *recording*

The *ex*-*vivo* somatosensory system preparation has been described in detail previously for adult mice [32,33]. We used a similar version of this same preparation in the current study in neonates. Briefly, male mice were anesthetized via injection of ketamine and xylazine (90 and 10 mg/kg, respectively) and perfused transcardially with oxygenated (95% O2-5% CO2) artificial CSF (aCSF; in mM: 1.9 KCl, 1.2 KH2PO4, 1.3 MgSO4, 2.4 CaCl2, 26.0 NaHCO3, and 10.0 D-glucose) containing 253.9 mM sucrose at approximately 12°C. The spinal cord and the right hindlimb was excised and placed in a bath of this aCSF. Hairy skin of the right hindpaw, saphenous nerve, L1-L5 DRGs and spinal cord were isolated. Following dissection, the preparation was transferred to a separate recording chamber containing chilled and oxygenated aCSF in which the sucrose was replaced with 127.0 mM NaCl. The skin was then pinned out on a stainless steel grid located at the bath/air interface, such that the dermal surface was allowed to be continuously perfused with the aCSF while the epidermis remained dry. The bath was then slowly warmed to 31°C before recording.

### Recording and stimulation

Sensory neuron somata within the L2 or L3 DRGs were impaled with quartz microelectrodes (impedance >150 MΩ) containing 5% Neurobiotin (Vector Laboratories, Burlingame, CA) in 1 M potassium acetate. Orthograde electrical search stimuli were delivered through a suction electrode placed on the side of the nerve to locate sensory neuron somata innervating the skin. Cutaneous receptive fields (RF) were localized with a soft brush and/or von Frey filaments. When cells were driven by the nerve but had no mechanical RF, a thermal search was conducted by applying hot (~52°C) and/or cold (~1°C) physiological saline to the skin. Although a concern may arise from this type of stimulation where brief but multiple applications of hot saline would result in sensitization of nociceptors during the course of an experiment, this has been thoroughly examined previously and has shown to not produce sensitization of other fibers recorded in the same preparations. That is to say, no differences were observed between fibers recorded at the beginning of an experiment compared to the end (e.g. [32,33,38]). Similar results were found here.

The response characteristics of individual DRG cells were then determined by applying mechanical and thermal stimuli to the hairy skin. For mechanical stimulation, RFs were probed with an increasing series of calibrated von Frey (VF) filaments ranging from 0.07 g to 10 g. When feasible, a mechanical stimulator that delivered a digitally controlled mechanical stimulus was also employed, which consisted of a tension/length controller (Aurora Scientific) attached to a probe with a 1 mm diameter aluminum tip. Computer controlled 5 s square waves of 1, 5, 10, 25, 50 and 100 mN were applied to the cell’s RF. In order to compare these results to those of the VF stimulation, VF units in grams were converted to a mN force based on the VF diameter. After mechanical stimulation, a controlled thermal stimulus was applied using a 3 mm^2^ contact area peltier element (Yale Univ. Machine Shop). Cold stimuli consisted of a variable rate cold ramp beginning at 31°C and reaching approximately 2-4°C, held for approximately 4-5 s and slowly allowed to return to 31°C. Bath temperature was maintained for a brief period and then the heat stimulus was initiated, which consisted of a 12 s heat ramp from 31-52°C followed by a 5 s plateau at 52°C. The stimulus then ramped back down to 31°C in 12 s. Adequate recovery times (approx. 20-30 s) were employed between stimulations. When recording from some myelinated sensory neurons, the heat ramp was continued to 54°C rather than 52°C and held for 5 s. In other instances, when fibers were unable to be fully characterized by controlled mechanical and thermal stimulation but were partially characterized by one of the controlled stimuli and brush or saline stimuli, these cells were also included for determination of afferent subtype prevalence and for the properties in which we obtained controlled data. All responses were recorded for offline analysis (Spike2 software, Cambridge Electronic Design). After physiological characterization, select cells were iontophoretically injected with 5% Neurobiotin (up to 2 cells per DRG). Conduction velocities of the recorded afferents were then calculated from spike latency and the distance between stimulating and recording electrodes (measured directly along the nerve). A total of 602 cells were intracellularly recorded and physiologically characterized in the current study. The average number of cells recorded per condition/time point/age was 54, which were obtained from an average of four mice per preparation. The minimum number of mice per preparation was three and an average of approximately 14 cells was recorded from each preparation. A minimum of 50 cells were obtained from each of the 11 groups analyzed. Specifically, the numbers of animals and cells recorded per condition were as follows: Naïve conditions: P7: 4 mice, 50 cells; P8: 6 mice, 54 cells; P10: 5 mice, 53 cells; P14: 5 mice, 58 cells; P21: 4 mice, 57 cells. Inflamed conditions: P7 inflammation: 1d: 4 mice, 57 cells; 3d: 3 mice, 52 cells; 7d: 4 mice, 56 cells; P14 inflammation: 1d: 3 mice 61 cells, 3d: 3 mice, 50 cells; 7d: 3 mice, 54 cells.

### Immunocytochemistry and analysis of single cells

After electrophysiological characterization and intracellular filling with Neurobiotin, the DRG containing the injected cell was removed and immersion fixed with 3% paraformaldehyde in 0.1 M phosphate buffer (PB) for 30 min at room temperature. Ganglia were then rinsed in 0.1 M PB, embedded in 10% gelatin, postfixed in 3% paraformaldehyde, and cryoprotected in 20% sucrose. 60 μm frozen sections were collected in PB from a sliding microtome (Thermo) and reacted with fluorescently-tagged (FITC) avidin to label Neurobiotin-filled cells (Vector Laboratories). Each section was also processed for ASIC3 (1:2000; Millipore; Cat#: AB5927; RRID: AB_92140) or TRPV1 (1:2000; Alomone; Cat# ACC-030; RRID: AB_2040256) immunoreactivity and isolectin B4 (IB4) binding (AlexaFluor 647; Molecular Probes). After incubation in primary antiserum, tissue was washed and incubated in appropriate fluorescently tagged secondary antibodies (1:200; Jackson Immunoresearch). Distribution of fluorescent staining was determined using Leica confocal microscope. Sequential scanning was performed to prevent bleed-through of the different fluorophores. Images were then captured and compiled using Adobe Photoshop.

### RNA isolation, reverse transcription and realtime PCR

Animals were first anesthetized as described above. The mice were then intracardially perfused with ice cold 0.9% NaCl prior to dissection of DRGs. RNA isolation from the L2 and L3 DRGs was performed using Qiagen RNeasy mini kits for animal tissues using the supplied protocol (n = 3-5 per condition/time point). RNA concentrations were then determined by obtaining A260 readings on a Nanodrop spectrometer (Thermo). Purified RNA was treated with DNase I (Invitrogen) and then DNased RNA was reverse transcribed using Superscript II Reverse Transcriptase (Invitrogen). For realtime PCR, 20 ng samples of cDNA were added to a SYBR Green Master Mix (Applied Biosystems) containing the appropriate primer combinations and run in duplicate on an Applied Biosystems Imager. Forward and reverse primer sequences used in realtime PCR reactions for ASIC1, ASIC3, GFRα3, TRPV1, TRPA1, trkA and GAPDH were obtained from Elitt et al. [71]. GFRα1, P2Y1 and P2X3 primer sequences were obtained from Jankowski et al. [32]. Primer sequences used for TRPC3, TRPM3, TRPM6, Piezo2, IGFr1 and IL1r1 are as follows: TRPC3: forward: 5′-AAG CAG GAT ATC TCC AGC CTT CGT-3′; reverse: 5′-AAG ATG GCT AAT TCC TCC GTC GCT-3′; TRPM3: forward: 5′-TTG AGG GAC CAG CTG TTG GT-3′; reverse: 5′-GTG CTG AGC TTG GGT TCG A-3′; TRPM6: forward: 5′-GTC TAC TGC CAT TCA GCC AAC CAA-3′; reverse: 5′-AGC CAA CAT CAG TTC TTC CAG GGT-3′; Piezo2: forward: 5′-AAG CCT TGG AAC TGG TGG TCT TCA-3′; reverse: 5′-ATA CCA TAG CCA GCC AAG AAG CCT-3′; IGFr1: forward: 5′-TTG AAC TTA TGC GCA TGT GCT GGC-3′; reverse: 5′-TCT CAT CCT TGA TGC TGC CGA TGA-3′; IL1r1: forward: 5′-AGG AAT GTG GCT GAA GAG CAC AGA-3′; reverse: 5′-ACT CGT GTG ACC GGA TAT TGC TTC-3′. Cycle time (Ct) values were normalized to GAPDH and changes in expression are calculated as a ΔΔCt value that is determined by subtracting the Ct values of the gene of interest from the GAPDH internal control for each sample and compared among samples. Fold change is described as 2^ΔΔCt^ (Applied Biosystems) and 2-fold change equals 100% change (mean ± SEM).

### Protein isolation and western blotting

Pooled L2/L3 DRGs from two naïve or peripherally inflamed mice were homogenized in lysis buffer containing 1% sodium dodecyl sulfate (SDS), 10 mM Tris–HCl (pH 7.4), and protease inhibitors (1 μg/ml pepstatin, 1 μg/ml leupeptin, 1 μg/ml aprotinin, 1 mM sodium orthovanadate and 100 μg/ml phenylmethylsulfonyl fluoride; Sigma Biochemicals). Then three to four of these pooled samples (15 μg) from each condition were centrifuged and boiled 10 min in a denaturing buffer containing β-mercaptoethanol and SDS prior to gel electrophoresis. Samples were then separated on a 12% polyacrylamide SDS-PAGE gel and transferred to a PVDF (Millipore) membrane that was blocked in specialized LiCor blocking buffer. Membranes were then incubated in primary antibodies overnight at 4°C (GFRα1: 1:250; R&D; Cat#: AF560; RRID: AB_2110307; GAPDH: 1:1000; ProSci Inc.; Cat#: XW-7214; RRID: AB_735758). Antibody binding was visualized using 680 nm or 800 nm infrared dye conjugated donkey anti-goat (Cat# 926–32214; RRID: AB_621846), or donkey anti-chicken (Cat# 926–32228; RRID: AB_1850018) secondary antibodies (1:15,000; LiCor) with detection using the LiCor Odyssey Imaging System (LiCor). Settings for detection were consistent between runs. Immunoreactive bands were analyzed by densitometry and quantified using NIH image J (RRID: nif-0000-30467) software. Band intensity was normalized to GAPDH and reported as a percent change (mean ± SEM).

### Data analysis

For electrophysiological analyses, one-way ANOVA tests and posthoc analysis (Tukey) or non-parametric, Kruskal-Wallis tests and posthoc analysis (Dunn’s test) were used to analyze data associated with mechanical and thermal thresholds and mean peak instantaneous frequencies (mean ± SEM) of both fast conducting and slowly conducting fibers [38,72]. This information was sorted by modality to examine whether certain subpopulations of sensory neurons have any consistency in regards to their response properties or expression of any of the markers used. In order to determine changes in the percentage of total fibers recorded among the multiple groups analyzed, a *χ*^2^ test was employed. In the few cases in which low numbers of afferents were obtained for a particular subpopulation, data were compared across conditions by also combining data from specific age groups to ascertain a generalized idea of the changes in response properties of afferents recorded during development or after inflammation. For a few ages, we obtained low numbers for only the mechanically sensitive A-fiber afferents that also responded to heat (APM), the small populations of mechanically insensitive but heat sensitive C-fibers (CH) and the polymodal C-fibers (CPM) that also responded to cold at certain ages/conditions (see Results above). Therefore in order to at least obtain a broad understanding of the potential changes in these afferent subtypes during the second and third weeks of development, data were combined for time points prior to P14 (P7-P10) and after P14 (P14-P21) and re-analyzed as described above. Combining data in this manner should allow us to determine general differences between the groups being analyzed as previous reports have shown that after approximately P7, no changes in response properties, particularly in A-fiber afferents, are typically detected in naïve animals [19,21,30]. Combining data in this manner was also performed from the time points post inflammation to more firmly state whether these particular subpopulations displayed altered response properties after peripheral injury. For mRNA or protein analyses, one-way ANOVA with Tukey’s post hoc assays were utilized. In order to determine the relative changes in gene expression that occur during postnatal development, all naïve time points were compared to L2/L3 DRGs obtained at P0 (n = 4). DRGs obtained after inflammation however, were compared to age-matched naïve controls. P-values were all set at p < 0.05.

## Competing interests

The authors declare no competing financial interests.

## Authors’ contributions

MPJ designed the experiments, performed the experiments, analyzed the data and wrote the paper. JLR, JDW, FBL, ATS and RCH performed the experiments and analyzed the data. All authors read and approved the final manuscript.
